# A sticky and heavily armed new species of *Solanum* (Solanumsubg.Leptostemonum, Solanaceae) from eastern Brazil

**DOI:** 10.3897/phytokeys.111.28595

**Published:** 2018-11-19

**Authors:** Yuri Fernandes Gouvêa, Leandro Lacerda Giacomin, João Renato tehmann

**Affiliations:** 1 Departamento de Botânica, Instituto de Ciências Biológicas, Universidade Federal de Minas Gerais – UFMG, Av. Antônio Carlos, 6627, Pampulha, Belo Horizonte, CEP 31270-901, MG, Brazil Universidade Federal de Minas Gerais Belo Horizonte Brazil; 2 Instituto de Ciências e Tecnologia das Águas & Herbário HSTM, Universidade Federal do Oeste do Pará, Av. Mendonça Furtado, 2946, Santarém, CEP 68040-050, PA, Brazil Universidade Federal do Oeste do Pará Santarém Brazil

**Keywords:** Neotropics, South America, Brazilian flora, spiny *Solanum*, new species, inselbergs, restinga, taxonomy

## Abstract

We describe a new species of spiny *Solanum* (Solanumsubg.Leptostemonum), endemic to the Brazilian Atlantic Forest and associated with granitic outcrops (inselbergs or sugar loaf mountains). *Solanumkollastrum* Gouvêa & Giacomin, **sp. nov.** is morphologically similar to the poorly known *S.sublentum* Hiern, but is a heavily armed, much more robust plant with stellate-glandular indumentum. Together with *S.sublentum*, it is morphologically related to some species of *Solanum* such as *S.hexandrum* Vell., *S.robustum* H.Wendl., and *S.stagnale* Moric. that share strongly accrescent calyces, large leaves with the bases decurrent on to the petiole, pendent simple inflorescences and large, robust flowers. The new species is restricted to a few known populations in southern Bahia and north-eastern Minas Gerais states and conservation efforts are needed.

## Abstract (pt)

Neste trabalho descrevemos uma nova espécie aculeada de *Solanum* (Solanumsubg.Leptostemonum) endêmica da Floresta Atlântica brasileira, associada a afloramentos graníticos (inselbergs ou pães de açúcar). *Solanumkollastrum* Gouvêa & Giacomin, **sp. nov.** é morfologicamente semelhante a *S.sublentum* Hiern, mas difere desta pelo hábito mais robusto e pelos ramos fortemente armados e recobertos por tricomas estrelados glandulares. Juntamente com *S.sublentum*, *S.kollastrum* é morfologicamente relacionada com algumas espécies sul-americanas que possuem cálices frutíferos fortemente acrescentes, folhas grandes com a base da lâmina decurrente no pecíolo, inflorescências pendentes simples e flores robustas, como *S.hexandrum* Vell., *S.robustum* H.Wendl. e *S.stagnale* Moric.. A espécie possui distribuição restrita ao sul da Bahia e nordeste de Minas Gerais, com poucas populações conhecidas, e necessita de esforços para conservação.

## Introduction

*Solanum* L., with about 1,400 species, is the most species-rich genus of the economically important Solanaceae family, in addition to being amongst the largest genera of flowering plants (Frodin 2004; Hawkes 1999). The distribution range of *Solanum* is proportional to its species richness: species belonging to the genus occur in all continents but Antarctica, with the highest diversity being found in tropical and subtropical regions of South America (Knapp 2002; Dupin et al. 2016). Phylogenetic studies have recovered the major lineages within *Solanum*, with the prickly species that possess stellate trichomes composing the largest of them, a monophyletic group known as the Leptostemonum clade or Solanumsubg.Leptostemonum Bitter (Bohs 2005, Levin et al. 2006, Weese and Bohs 2007, Särkinen et al. 2013). The ‘spiny solanums’, as the group is informally named, comprises two major groups: the Old World lineage, which is particularly diversified in Australia, eastern Africa (incl. Madagascar) and tropical Asia and the highly diverse New World grade, that includes some Torva and Lasiocarpa clade representatives native to both the New and Old Worlds (Stern et al. 2011; Vorontsova et al. 2013; Aubriot et al. 2016).

Brazil, especially the eastern portion of its territory, is one of the primary centres of diversity and endemism for both non-spiny (Knapp 2002) and spiny solanums (Whalen 1984) in the New World. With approximately 110 species of spiny solanums (Agra 2007; BFG 2015) and 10 of the 13 New World lineages recovered in Stern et al. (2011), the Brazilian *Solanum* flora is exceedingly diverse. Ongoing efforts to document and describe the diversity of *Solanum* in the country through modern taxonomy and intensive fieldwork efforts (i.e. Flora do Brasil 2020 project; http://floradobrasil.jbrj.gov.br/, also see BFG 2015) have shed light on the taxonomy of endemic groups (e.g. Asterophorum clade, Gouvêa and Stehmann in press.; Inornatum clade, Giacomin 2015) and led to the discovery of various undescribed species (e.g. Giacomin and Stehmann 2014; Knapp et al. 2015; Gouvêa and Stehmann 2016; Agra and Stehmann 2016). Here we describe a new species of spiny *Solanum* from the states of Bahia and Minas Gerais associated with granitic outcrops (inselbergs), a poorly sampled environment with a high degree of endemism in many plant groups (Martinelli 2007, Porembski 2007, de Paula et al. 2017).

## Material and methods

For the present study, specimens from the following herbaria were examined: ALCB, BHCB, CEPEC, FURB, HUEFS, MBM, MBML, NY, RB, RFFP and UFP (acronyms from Index Herbariorum; http://sweetgum.nybg.org/science/ih). Expeditions to the areas where the new species occurs were carried out in June 2014, September 2015 and June 2018. During these expeditions, in addition to herbarium collections, juvenile plants were also collected *in situ* and cultivated at the Museu de História Natural e Jardim Botânico da Universidade Federal de Minas Gerais to obtain further information about plant development and morphology. Measurements of reproductive characters were performed in both dry and fresh or fixed (70% alcohol) material. Terminology used to describe the overall morphology and indumentum follows Radford et al. (1976), but trichome typology was based on Roe (1972) and Mentz et al. (2000). Conservation status was assessed using the IUCN Red List Categories and Criteria (IUCN 2017) and for that, estimates of extent of occurrence (EOO) and area of occupancy (AOO) were calculated using the GeoCat tool (www.geocat.kew.org; Bachman et al. 2011) with the cell size of 2 km^2^ for AOO. The criteria used for species delimitation is based on the morphological cluster species concept (Mallet 1995).

## Taxonomic treatment

### Solanum kollastrum

Taxon classificationPlantaeSolanalesSolanaceae

Gouvêa & Giacomin sp. nov.

urn:lsid:ipni.org:names:77192011-1

**Figure 1.**
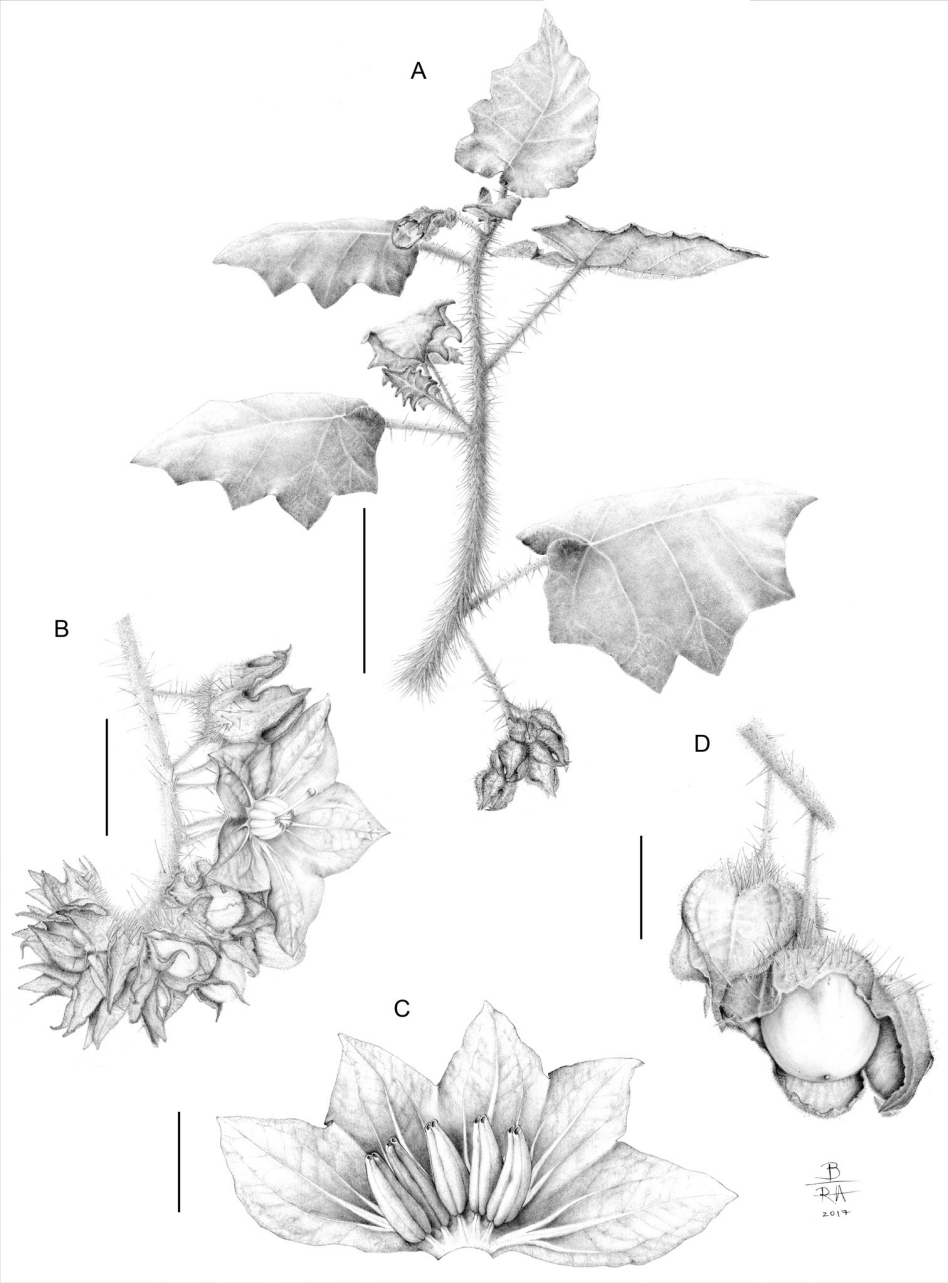
Line drawing of *Solanumkollastrum*. **A** habit with notably dense prickles, leaves lacking secondary lobes and internodal inflorescences **B** detail of the prickly inflorescence with a hermaphrodite flower at anthesis **C** detail of a dissected flower **D** detail of the fruits enclosed by the strongly accrescent fruiting calyces (with the uppermost fruiting calyx opened to expose the fruit) from *Gouvêa 102* (BHCB).

**Figure 2.**
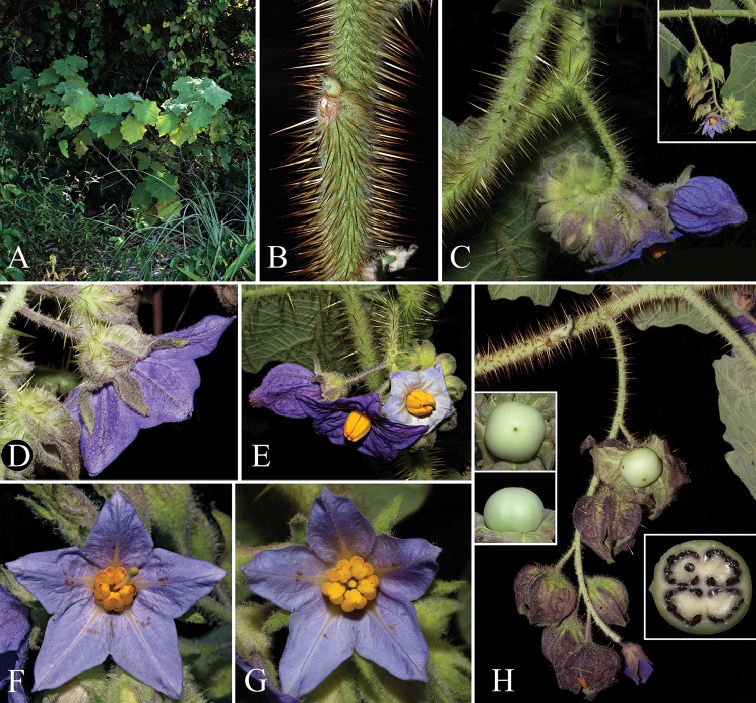
*Solanumkollastrum*. **A** plant habit **B** detail of stem prickles **C** young inflorescence (upper right corner: detail of a more developed inflorescence) **D** flowering calyx **E** a short-styled and a long-styled flower displaying the extremes of variation of corolla size and colour found in the species (here exhibited by flowers of the same inflorescence); also note the various degrees of anther curvature and location of the apical pores compared with the images F and G **F** long-styled flower (hermaphroditic) with extrorse pores and slightly outwardly curved apices **G** short-styled flower (functionally male) with extrorse pores and markedly outwardly curved apices **H** infructescence with details of the strongly accrescent fruiting calyces (one of which was dissected to show the fruit) and the mature fruit colour (left side: details of fruit shape; right side: detail of a dissected fruit showing the four locules, placentation, seed colour and shape). Scale bars: 1.2 m (**A**); 3 cm (**B, C, H**); 1.5 cm (**D, F, G**); 1.8 cm (**E**). Photographs by Y.F. Gouvêa.

**Figure 3.**
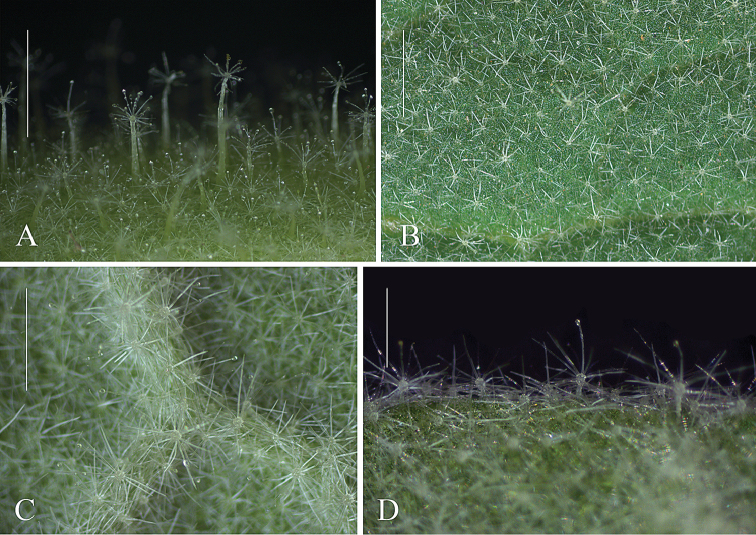
Detail of *Solanumkollastrum* indumentum. **A** trichomes composing the indumentum of the stems, petioles and inflorescence axis **B** indumentum of the adaxial leaf surface **C** Indumentum of the abaxial leaf surface **D** Trichomes composing the indumentum of the abaxial leaf surface. Scale bars: 6 mm (A); 5 mm (B); 3 mm (C); 1.5 mm (D). Photographs by Y.F. Gouvêa

Figures 1 , 2 , 3 , 5

#### Diagnosis.

Differs from *S.sublentum* Hiern in its tomentose young stems, petioles and inflorescence axis with the indumentum composed of long-stalked (up to 1 cm) stellate-glandular trichomes with all rays glandular (versus pubescent-glandular indumentum composed of persistent simple glandular and persistent to early deciduous sessile to short-stalked stellate trichomes with only the midpoint glandular), in its straight stem prickles up to 17 mm long (versus recurved to oblique stem prickles up to 6 mm long) and in its large mature leaves 20.5–42 cm long and 20–38 cm wide (versus mature leaves 5.7–17 cm long and 3.8–14 cm wide).

#### Type.

Brazil. Minas Gerais: Ataléia, povoado de Canaã do Brasil, estrada não pavimentada que liga o município de Ouro Verde de Minas ao povoado de Canaã do Brasil, crescendo em área alterada próximo a afloramento rochoso gnáissico (inselberg ou pão de açucar), 18°00'19"S, 41°12'17"W, 313 m elev., June 2018 (fl, fr), *Y.F Gouvêa 280* (holotype: BHCB [BHCB190863]; isotype: RB).

#### Description.

Shrubs up to 3.5 m, erect, moderately branched. Young stems terete, densely tomentose with hyaline to ochraceous stellate-glandular trichomes, these sessile to long-stalked with multiseriate stalks up to 1 cm long, multiangulate, the rays 5–20, 2–3-celled, unequal in length, all or almost all with a capitate glandular distal cell, the midpoint 2–3-celled, equal to or twice the length of the longest ray, the distal cell glandular; stems densely armed with prickles up to 17 mm long and to 2.3 mm wide at the base, straight, slightly flattened, stramineous to yellowish at base, becoming ferruginous towards the apex, pubescent with stellate trichomes like those of the stems and some small, stalked, uniseriate glandular trichomes at the base; bark of older stems greyish dark brown. Sympodial units difoliate to plurifoliate, the leaves not geminate, the leaves arranged in a 2/5 phyllotaxic spiral. Leaves simple, lobed, 20.5–42 cm long, 20–38 cm wide, the blade broadly elliptic to broadly ovate, membranous, discolorous, green adaxially and whitish light green abaxially when fresh, becoming dark green adaxially and light green to pale brown abaxially when dried; adaxial surface densely stellate-glandular tomentose but always visible, with multiangulate trichomes, these short- to long-stalked, with multiseriate stalks 3–4 cells wide, up to 1 mm long, the rays 4–11, 1-celled, all eglandular or with one or more glandular ones (then 2–3-celled), unequal in length, the midpoints 2–3-celled, usually longer than the rays, mixed with smaller porrect to antrorse, usually eglandular stellate trichomes, these sessile to short-stalked (stalks to 0.1 mm long), the rays 2–5, 1-celled and minute, inconspicuous, unbranched, subsessile uniseriate glandular trichomes; the abaxial surface densely stellate-glandular tomentose, the epidermis barely visible, with trichomes like those of the adaxial surface, but more densely distributed; sparsely to moderately armed along the midrib and the primary veins of both surfaces with straight, laterally compressed prickles reaching up to 10 mm long and to 1.3 mm wide at the base adaxially, up to 17.5 mm long and to 1.8 mm wide at the base abaxially; primary veins 5–7 pairs; base cordate, the two major basal lobes obtuse to rounded, 2.5–7 cm long at the longest point, often overlapping each other over the petiole, not decurrent on to the petiole; margins with the lateral lobes 1.5–4.8 cm long, 4–9 cm wide at base, acute or less often obtuse or rounded at the apex, both basal and lateral lobes sometimes with small secondary lobes; apex acute; petiole 4.5–19.5 cm, densely tomentose with trichomes like those of the stem, armed. Inflorescence a scorpioid cyme, usually unbranched, rarely forked or trifurcate, internodal or subopposite the leaves, the axis densely glandular tomentose with trichomes like those of the stem, but these hyaline to ochraceous, armed; peduncles 2.6–6 cm long, the rachis 4.3–11 cm long, with 11–35 flowers, with up to 3 open at the same time; pedicel insertions generally unequally spaced, adjacent to spaced 2.3 cm apart; pedicels 4.8–18 mm long in open flowers, straight, articulated at base, armed, densely tomentose with trichomes like those of the stem, but with the epidermis and trichomes often purple-coloured. Flowers 5-merous, the plants andromonoecious, producing hermaphroditic flowers (long-styled) and functionally male short-styled flowers, which vary in proportion (number of long- vs short-styled flowers) between inflorescences. Calyx somewhat urceolate, inflated, foliaceous, purple (mainly along the margins and apex of the calyx lobes) to green, armed, densely tomentose with the epidermis barely to not visible basally, becoming gradually more visible towards the apex of the lobes, with trichomes like those of the stem but these sometimes purple and with some eglandular rays; base rounded, markedly plicate on the fusion line at the base of the adjacent sepals, these basally concave, the calyx tube 4.5–8.2 mm long, 9.4–15.2 mm in diameter at the point with the largest diameter, the lobes 7.5–15.6 mm long, 6–9 mm wide at the base, triangular, the margins plane to strongly undulate and revolute, the apices acute to caudate. Corolla 2.3–3.9 cm in diameter, purple to lilac or bluish-lilac, stellate, lobed 2/5 to 1/2 of its length, interpetalar tissue absent, the tube 1.1–2.2 cm long, the lobes 10.9–15 mm long, 8.8–13.4 mm wide, deltate to triangular, the margins straight to slightly convex at base, the apex acute, apiculate or not, stellate-glandular tomentose abaxially with trichomes like those of the leaves, almost glabrous adaxially with trichomes sparsely distributed along the veins and near the apex. Stamens equal; filament tube 1–2.1 mm long; free portion of the filaments 1.3–2.9 mm long, glabrous; anthers 7.5–10 mm long, 2.8–4.3 mm wide, 2.4–2.9 mm thick at the widest point, slightly gibbous, broadly lanceolate, narrowed towards the apex, sagittate at base, connivent, with the pores directed to apex and slightly extrorse, the epidermis papillose, slightly swollen dorsally. Ovary conical to somewhat cupuliform, 4-lobed, 4-locular, densely stellate-glandular tomentose at the apex, becoming glabrous with age, the trichomes 2–7-rayed, stellate, sessile, with a 2–4-celled, eglandular or glandular midpoint longer than the 1-celled rays; style 13.7–15.9 mm long in long-styled flowers, 1.2–3.7 mm long in short-styled flowers, cylindrical, glabrous; stigma globose to clavate, up to 1.4 mm long in long-styled flowers, papillose, green when fresh. Infructescence axis up to 29 cm long. Fruit a widely depressed ovoid to obloid berry, 11.4–20 mm long, 12–22.5 mm wide, the pericarp smooth, pale green to white, with scattered stellate trichomes at the apex; fruiting pedicels 1.4–2.2 cm long, armed; fruiting calyx strongly accrescent, completely covering the fruit in all stages of development, the tube 16–20.4 mm long and 19–34 mm in diameter at the widest point, the lobes 11–21.8 long, 13.7–19 mm wide at base. Seeds ca. 230 per berry, ca. 2 mm long and 2.4 mm wide, flattened, reniform, dark brown. Chromosome number: not known.

#### Distribution.

**Figure 4.**
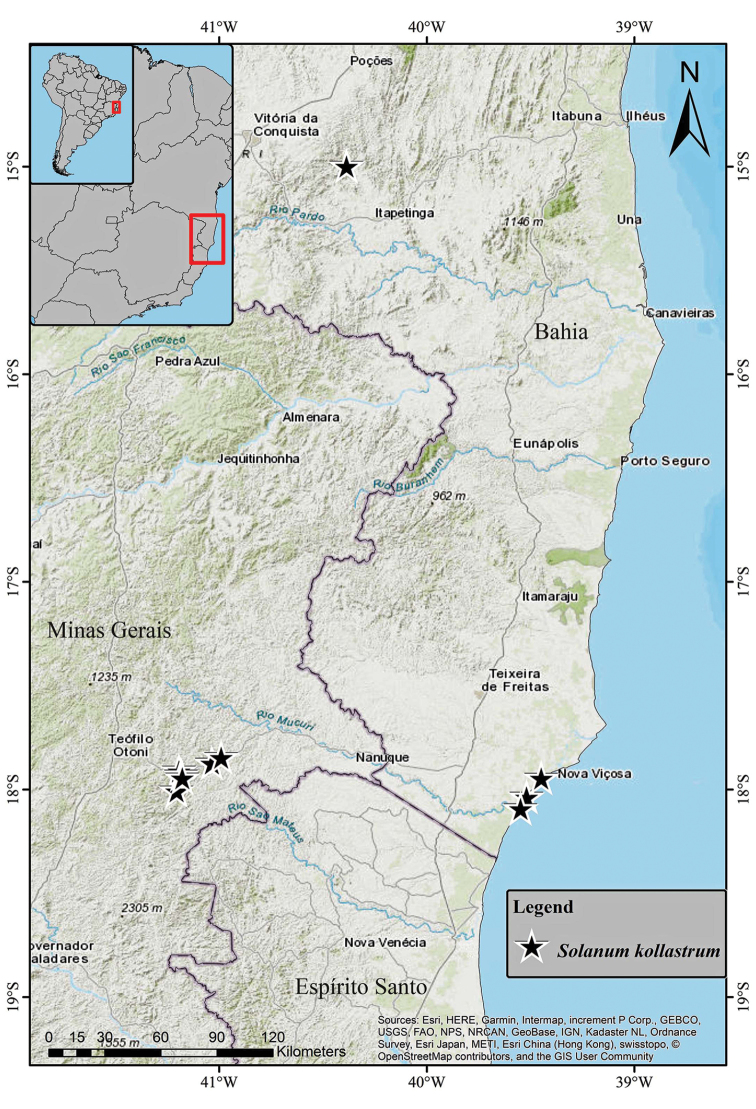
Distribution of *Solanumkollastrum*.

Endemic to eastern Brazil (Figure 4). The known records of *Solanumkollastrum* are mostly concentrated along the Mucuri River watershed, ranging from the municipality of Ataléia, in northeastern Minas Gerais state, to Mucuri at the southern coast of Bahia. The only exception, so far, is one collection (*J.G. Jardim et al. 3151*; CEPEC, NY) made further north, in Caatiba, a municipality of the south-central region of Bahia State.

#### Ecology and habitat.

*Solanumkollastrum* inhabits the edge of small forest fragments, especially those at the base or on granitic outcrops (inselbergs), which are geological formations characterising the peculiar landscape of the type locality. Some populations were also found in disturbed sites near these rock outcrops, such as borders of unpaved roads and pastures. The restinga (herbaceous to arboreal vegetation growing along the Brazilian sandy coastal lowlands; Araújo 1992) is a most distinct environment in which *S.kollastrum* has been found [*S.A. Mori et al. 10459* (CEPEC, NY), *Y.F. Gouvêa 283* (BHCB) and *Y.F. Gouvêa 284* (BHCB)]. In restinga formations, *S.kollastrum* was observed in open disturbed areas dominated by grasses and at the edge of forest fragments near the Mucuri River mouth in Bahia State (Fig. 4). The known *S.kollastrum* habitats vary from environments subject to periods of drought (e.g. the edge of small seasonal semi-deciduous forest fragments or vegetation islands on inselbergs) to constantly wetter environments, at the edge of the aforementioned coastal forests, where the climate is under a strong oceanic influence. Its observed elevational range is from sea level to about 900 m. Field observations as well as its anther morphology (i.e. poricidal dehiscence and the anthers’ robustness) suggest that its primary pollinators are medium- to large-sized bees (e.g. genus *Ptiloglossa*) with buzzing behaviour (Michener 1962, Buchmann 1983). *Solanumkollastrum* fruits hang outside (below) the foliage on long inflorescence axes and are enclosed until their maturity by an inflated *Physalis*-like calyx. This, along with the persistent green to white epicarp colour, the fleshy mesocarp, the numerous relatively small seeds and the release of a mild sweetish scent at fruit maturity are characteristics associated with fruits eaten by bats (Van der Pijl 1972, Cooper et al. 1986, Charles-Dominique and Cockle 2001). Actually, several studies have shown the importance of the fruits of *Solanum* species in bat diets (Marinho-Filho 1991, Passos et al. 2003, Zanon and dos Reis 2007, Mello et al. 2008a) and the role of bats as dispersal agents for *Solanum* species (Uieda and Vasconcellos-Neto 1985, Iudica and Bonaccorso 1997, Galindo-Gonzáles et al. 2000, Mello et al. 2008b). Many other *Solanum* species also present such features (e.g. those here considered morphologically related to *S.kollastrum*; see discussion). However, species-level studies on pollination or fruit dispersal of Brazilian *Solanum* species are virtually non-existent, although being fundamental to confirm and better understand the interactions between these species and their pollinators and dispersal agents.

#### Phenology.

Flowering specimens were found from April to November, when immature fruits were also observed, indicating that *Solanumkollastrum* may bloom throughout most of the year. Specimens with mature fruits were observed at the end of June.

#### Etymology.

The epithet *kollastrum* is derived from the Greek words for glue (κόλλα) and star (άστρον), referring to the notable stellate-glandular trichomes observed on the younger stems, petioles and inflorescence axis of this species.

#### Preliminary conservation status.

Endangered (EN) B2 a, b (ii, iii, iv); Extent of Occurrence (EOO) 32,626 km^2^ (NT); Area of Occupancy (AOO) 20 km^2^ (EN). Despite the relatively large EOO (>20,000 km^2^) of *S.kollastrum*, its small AOO (<500 km^2^), the few and disjunct collections, all outside protected areas and the vulnerability of its habitats, lead us to suggest it should be attributed an Endangered status (IUCN 2017).

#### Additional specimen examined (paratypes).

**BRAZIL. Bahia**: Mun. Mucuri, Rodovia Mucuri/Nova Viçosa (BA-001), crescendo em área de restinga aberta alterada dominada por gramíneas à margem da rodovia, 18°02'08"S, 39°31'10"W, 3 m elev., June 2018 (fl, fr), *Y.F. Gouvêa 283* (BHCB); a 4 km a W de Mucuri, Restinga, 13 September 1978 (fl), *S.A. Mori et al*. *10459* (CEPEC, NY); Mun. Nova Viçosa, Rodovia Mucuri/Nova Viçosa (BA-001), crescendo em borda de fragmento de restinga arbórea à margem da rodovia, 17°56'37"S, 39°26'54"W, 5 m elev., June 2018 (fl, fr), *Y.F. Gouvêa 284* (BHCB); Mun. Caatiba, entrada para a cidade ca. 11 km de Itapetinga, rod. para Caatiba 31.2 km da BR-415, 14°59'48"S, 40°23'12"W, 427 m elev., 3 November 2000 (fl, fr), *J.G. Jardim et al. 3151* (CEPEC, NY). **Minas Gerais**: Mun. Ataléia, estrada de terra que leva da BR-418 à comunidade Canaã, 17°56'34"S, 41°10'39"W, 382 m elev., 15 June 2014 (fl, fr), *Y.F. Gouvêa et al*. *102* (BHCB); Mun. Teófilo Otoni, Rodovia BR-418, crescendo à sombra entre rochas da base de afloramento rochoso gnáissico (inselberg ou pão de açúcar) à margem da rodovia, 17°54'33"S, 41°11'37"W, 225 m elev., June 2018 (fl, fr), *Y.F. Gouvêa 281* (BHCB); Pedra da Boca, topo do inselberg, crescendo na borda de capão de mata, 17°55'44.18"S, 41°11'1.36"W, 911 m elev., 20 September 2015 (fl, fr), *J.R. Stehmann et al*. *6387* (BHCB); Mun. Carlos Chagas, Rodovia BR-418, crescendo em área alterada no entorno de afloramento rochoso gnáissico (inselberg ou pão de açúcar) próximo à margem da rodovia, 17°52'16"S, 41°02'07"W, 280 m elev., June 2018 (fl, fr), *Y.F. Gouvêa 282* (BHCB); Rod. BR-418, km 112, base dos paredões rochosos, 11 April 1984 (fl, fr), *G. Hatschbach 47806* (CEPEC, NY).

## Discussion

*Solanumkollastrum* is morphologically related to a group of species endemic to the south-eastern Brazilian Atlantic Forest that share strongly accrescent fruiting calyces, large leaves with decurrent bases and large, robust flowers (see Fig. 2). This unnamed group appears to include five known species (i.e. *S.hexandrum* Vell., *S.kollastrum*, *S.robustum* H.Wendl., *S.stagnale* Moric., and *S.sublentum*). Of these, three species (*S.hexandrum*, *S.robustum* and *S.stagnale*) were sampled in the molecular phylogeny of Stern et al. (2011), forming a moderately supported clade sister to the clade that includes species traditionally placed in SolanumsectionErythrotrichum A.Child. Although Stern et al. (2011) included both of these groups in the Erythrotrichum clade, the lineage containing *S.kollastrum* and related species may deserve recognition as a separate clade, emphasising its morphological, ecological and geographical distinctive nature.

**Figure 5.**
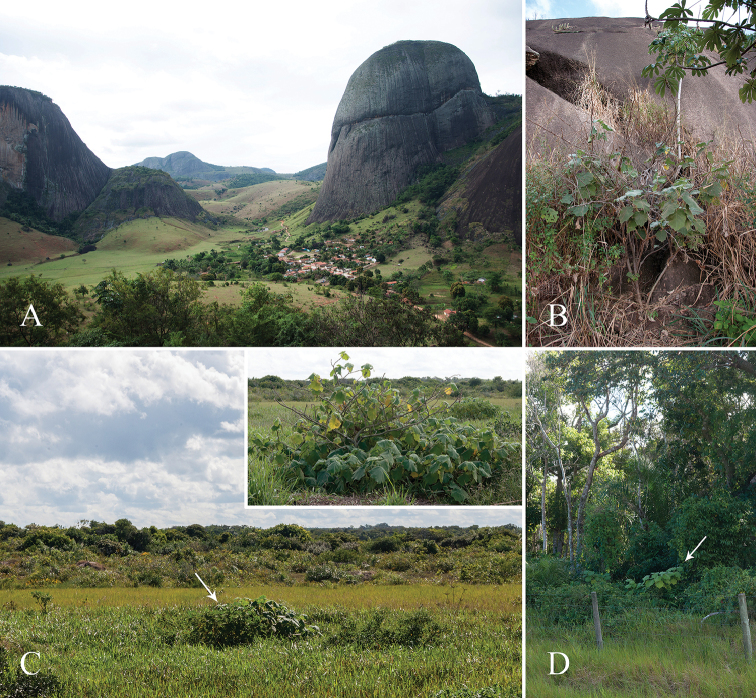
Habitats of *Solanumkollastrum*. **A** general view of the small village of Canaã do Brasil illustrating the typical landscapes of the type locality **B** a specimen growing in the soil amongst rocks at the base of an inselberg **C** general view of an area of altered restinga vegetation with the arrow pointing to a *S.kollastrum* specimen (upper right corner: detail of the distinct architecture of the specimens growing in this environment) **D** a specimen growing at the edge of a restinga forest fragment. Photographs by Y.F. Gouvêa

Amongst the species in this group, only *S.kollastrum* and *S.sublentum* have glandular trichomes on the entire plant (see Fig. 3) and cordate leaf bases. Decurrent leaf bases of *S.kollastrum* are only seen in the first leaves of the seedlings, with the subsequent leaves gradually changing shape to become cordate and non-decurrent. In contrast, the leaf bases in *S.hexandrum*, *S.robustum* and *S.stagnale* remain decurrent throughout the plants’ life, varying in shape from attenuate to truncate. *Solanumkollastrum* most closely resembles *S.sublentum*, of which it can be readily distinguished by the robust long-stalked (up to 1 cm) stellate-glandular trichomes with all rays having a glandular distal cell (some rays may lose the glandular cell through breakage or by the disruption of the gland wall) composing the indumentum of its young stems, petioles and inflorescence axis (see Fig. 3A; trichomes in *S.sublentum* are mostly simple). The shape and length of its stem prickles and the robustness of its leaves also are useful for the distinction between these species (see diagnosis for more details). Although easily differentiated, *Solanumkollastrum* and *S.sublentum* have very similar floral morphologies, sharing well-developed calyces that are strongly accrescent in fruits, showy purple to lilac corollas and robust anthers (see Fig. 2). Their leaves also resemble each other: both are lobed (with secondary lobes or not), elliptic to ovate (or broadly ovate in *S.kollastrum*) and have cordate bases (varying from truncate to cordate or sagitate in *S.sublentum*). In addition, the glandular nature of their trichomes, although they differ in type, is also a common character to both species. In the field, *S.kollastrum* has notably larger leaves than those of *S.sublentum*, however, usually only the apices of the branches are collected, with the fully developed leaves not represented in herbarium material, so this character is often not apparent from herbarium specimens. The diameter of the stems at the middle portion of the internode between the two youngest mature leaves is, in most cases, an additional distinguishing character between these species (0.9–3 mm in *S.sublentum* and 3.8–6.6 mm in *S.kollastrum*). Despite the fact that *S.kollastrum* and *S.sublentum* occur in similar environmental conditions (associated with outcrops or at edges of lowland forests, see Fig. 5), they have not been observed in sympatry.

The size and colour of the *S.kollastrum* corollas, the shape of its anthers and density and shape of its stem prickles are quite variable. The corollas of the examined specimens vary from 2.3 to 3.9 cm in diameter and from purple to bluish-lilac, with flowers exhibiting sometimes the extremes of variation of these characters in the same inflorescence (see Fig. 2E). The anther shape and the position of the apical pores also varies considerably; the anthers of plants from the type locality are straight (typical) with slightly extrorse apical pores, while those of plants from the coastal region have apices with varying degrees of curvature outwards from the cone, with pores markedly extrorse (see Figs 2E–G). Despite the observed variability, apically curved anthers are only found in *S.kollastrum* when compared to morphologically similar species. This distinct anther morphology may reflect differences in the plant-pollinator interaction, being an interesting issue for further investigation. The length of trichome rays and midpoints is also variable; plants collected in Caatiba, Bahia, have stem, petiole and inflorescence axis trichomes with rays and midpoints much longer than those specimens from other localities.

Plants growing in open restinga vegetation sites exhibit distinct architecture. These plants are lower and wider in their overall appearance due to the branching near the base of the major stem, with which the first order branches form angles close to 90° (see Fig. 5C). Plants from other habitats are more erect and become taller, with the first order branches forming angles close to 45° (habit with Y-shaped overall appearance; see Figs 5B, 5D and 2A). Differences in density and shape of the stem prickles between populations from inland and coastal areas is also observed, with the coastal populations possessing moderately distributed stem prickles with slightly broader bases, rather than the densely distributed narrowly based needle-like prickles of inland populations.

The discovery of *S.kollastrum*, a robust and conspicuous plant growing at the roadsides in regions close to large urban centres, highlights how insufficiently known the Brazilian flora is, even at present, and how urgent the need is to describe, study and conserve the country’s plant diversity. Thus, we hope that this discovery encourages the study on the most diverse aspects of this species’ biology.

## Supplementary Material

XML Treatment for Solanum kollastrum
